# Post‐COVID dysphagia requires exclusion of SARS‐CoV‐2–associated brainstem encephalitis, vasculitis, polyneuritis cranialis, and myositis

**DOI:** 10.1002/brb3.70032

**Published:** 2024-09-24

**Authors:** Josef Finsterer

**Affiliations:** ^1^ Neurology & Neurophysiology Center Vienna Austria

We read with interest the article by Kim et al. about three patients with dysphagia 7–14 days after SARS‐CoV‐2 infection (SC2I) and elevated antibodies against GD1b (patient‐1), GQ1b (patient‐2), and GM1 (patient‐3) suggesting a Guillain–Barre syndrome (GBS) subtype (Kim et al., 2024). Comprehensive rehabilitation with speech therapy led to only slight (patient‐1), no (patient‐2), or significant improvement in dysphagia as assessed by FEES after 7 months (patient‐1), 4 weeks (patient‐2), and 4 months (patient‐3) of the follow‐up (Kim et al., 2024). The study is impressive, but some points require further discussion.

The first point is that the dysphagia in the three patients was explained either by the presence of GBS or by cross‐reactivity between anti‐ganglioside antibodies and SARS‐CoV‐2 spike protein (Kim et al., 2024), but it remained unclear by which criteria GBS was diagnosed. Was the diagnosis GBS made using the Brighton criteria (Fokke et al., 2014), the Leonhard criteria (Leonhard et al., 2019), or the EAN criteria (van Doorn et al., 2023)?

The second point is that myositis has not been discussed as a possible cause of dysphagia. It is known that myositis is a common complication of SC2I, and myositis can manifest as dysphagia (Aoyagi et al., 2021).

The third point is that it remained unclear whether magnetic resonance imaging (MRI) was performed with or without contrast medium. Brainstem encephalitis or radiculitis of the lower cranial nerves as the cause of dysphagia can only be ruled out by an MRI with contrast medium (Dukkipati et al., 2022).

The fourth point is that cerebral vasculitis has not been discussed as a possible cause of dysphagia. Several cases of SC2I‐associated cerebral vasculitis have been reported (Rhodes et al., 2024), and cerebral vasculitis can be complicated by dysphagia. One method of diagnosing cerebral vasculitis is imaging with contrast medium and angiography.

The fifth point is that patient‐2 had elevated GQ1b antibodies, which is pathognomonic for Miller–Fisher syndrome. Therefore, we should know whether there was evidence of ataxia, areflexia, or ophthalmoparesis in this particular patient. It is also imperative to consider the pharyngeal‐cervical‐brachial variant of GBS (Randhawa et al., 2021).

The sixth point is that patient‐1 already had diabetes and that diabetic cranial neuropathy could not be sufficiently ruled out. Since patients with pre‐existing polyneuropathy can more easily develop GBS with more severe manifestations, it would have been important to know whether diabetes was well controlled or whether HbA1c values were significantly elevated. What was the cause of demyelinating polyneuropathy in patient‐2? Was it GBS?

The seventh point is that patient‐1 required mechanical ventilation, but it was not taken into account that the respiratory failure could be due to respiratory muscle involvement in GBS and not necessarily SC2I‐related pneumonia.

We disagree with the statement in the abstract that the three patients had isolated dysphagia. Since documented polyneuropathy was present in at least two patients (Kim et al., 2024), there was also at least subclinical involvement of peripheral nerves.

We also disagree with the statement in the limitations that the included patients had “no significant motor weakness” at presentation (Kim et al., 2024). Since all three patients had dysphagia and dysphagia can also be caused by the involvement of the striated pharyngeal muscles, motor involvement cannot be ruled out at the onset of the disease.

In conclusion, patients with post‐COVID dysphagia require a comprehensive workup for brainstem encephalitis and vasculitis, cranial polyneuritis, polyneuropathy, and myositis.

## AUTHOR CONTRIBUTIONS

**Josef Finsterer**: Investigation; conceptualization; data curation; formal analysis; writing—review and editing; writing—original draft.

## CONFLICT OF INTEREST STATEMENT

The author declares no conflicts of interest.

### PEER REVIEW

The peer review history for this article is available at https://publons.com/publon/10.1002/brb3.70032.

## Data Availability

Data sharing not applicable to this article as no datasets were generated or analyzed during the current study.

## References

[brb370032-bib-0001] Aoyagi, Y. , Inamoto, Y. , Shibata, S. , Kagaya, H. , Otaka, Y. , & Saitoh, E. (2021). Clinical manifestation, evaluation, and rehabilitative strategy of dysphagia associated with COVID‐19. American Journal of Physical Medicine & Rehabilitation, 100(5), 424–431. 10.1097/PHM.0000000000001735. 33657028 PMC8032217

[brb370032-bib-0002] Dukkipati, S. S. , Zhou, D. J. , Powers, A. M. , Piccione, E. A. , & Koh, S. (2022). Acute bulbar palsy‐plus variant of Guillain‐Barré syndrome in a 3‐year‐old girl. Child Neurology Open, 9, 2329048X2211154. 10.1177/2329048X221115476 <./bib> PMC9350509 35936111

[brb370032-bib-0003] Fokke, C. , Van Den Berg, B. , Drenthen, J. , Walgaard, C. , Van Doorn, P. A. , & Jacobs, B. C. (2014). Diagnosis of Guillain‐Barré syndrome and validation of Brighton criteria. Brain, 137(Pt 1), 33–43. 10.1093/brain/awt285. 24163275

[brb370032-bib-0004] Kim, S. , Bae, J. , Park, G.‐Y. , & Im, S. (2024). Implications of anti‐ganglioside antibodies in isolated dysphagia following COVID‐19 infection: Case series. Brain and Behavior, 14(5), e3514. 10.1002/brb3.3514. 38698593 PMC11066413

[brb370032-bib-0005] Leonhard, S. E. , Mandarakas, M. R. , Gondim, F. A. A. , Bateman, K. , Ferreira, M. L. B. , Cornblath, D. R. , Van Doorn, P. A. , Dourado, M. E. , Hughes, R. A. C. , Islam, B. , Kusunoki, S. , Pardo, C. A. , Reisin, R. , Sejvar, J. J. , Shahrizaila, N. , Soares, C. , Umapathi, T. , Wang, Y. , Yiu, E. M. , … Jacobs, B. C. (2019). Diagnosis and management of Guillain‐Barré syndrome in ten steps. Nature Reviews Neurology, 15(11), 671–683. 10.1038/s41582-019-0250-9. 31541214 PMC6821638

[brb370032-bib-0006] Randhawa, J. , Randhawa, H. S. , & Toor, P. (2021). Pharyngeal‐cervical‐brachial variant of Guillain‐Barré syndrome in a patient of COVID‐19 infection. Cureus, 13(9), e17945. 10.7759/cureus.17945<./bib> 34548992 PMC8437512

[brb370032-bib-0007] Rhodes, R. H. , Love, G. L. , Da Silva Lameira, F. , Sadough Shahmirzadi, M. , Fox, S. E. , & Vander Heide, R. S. (2024). Acute neutrophilic vasculitis (leukocytoclasia) in 36 COVID‐19 autopsy brains. Diagnostic Pathology, 19(1), 33. 10.1186/s13000-024-01445-w . 38360666 PMC10870569

[brb370032-bib-0008] Van Doorn, P. A. , Van Den Bergh, P. Y. K. , Hadden, R. D. M. , Avau, B. , Vankrunkelsven, P. , Attarian, S. , Blomkwist‐Markens, P. H. , Cornblath, D. R. , Goedee, H. S. , Harbo, T. , Jacobs, B. C. , Kusunoki, S. , Lehmann, H. C. , Lewis, R. A. , Lunn, M. P. , Nobile‐Orazio, E. , Querol, L. , Rajabally, Y. A. , Umapathi, T. , … Willison, H. J. (2023). European Academy of Neurology/Peripheral Nerve Society Guideline on diagnosis and treatment of Guillain‐Barré syndrome. European Journal of Neurology, 30(12), 3646–3674. 10.1111/ene.16073. 37814552

